# The Implications of 9th Lung Cancer TNM Classification on Interventional Pulmonology

**DOI:** 10.1002/resp.70243

**Published:** 2026-03-24

**Authors:** Judith Maria Brock, Daniela Gompelmann

**Affiliations:** ^1^ Department for Pneumology and Critical Care Medicine Thoraxklinik at University of Heidelberg Heidelberg Germany; ^2^ Translational Lung Research Center Heidelberg (TLRC) German Center for Lung Research (DZL) Heidelberg Germany; ^3^ Division of Pulmonology, Department of Internal Medicine II Medical University of Vienna Vienna Austria; ^4^ Comprehensive Cancer Center Medical University of Vienna Vienna Austria

**Keywords:** bronchoscopy, endobronchial ultrasound, lung cancer, lymph node staging, TNM

The implementation of the 9th edition of the TNM classification for lung cancer, effective January 2025 and revised by the International Association for the Study of Lung Cancer, introduces critical refinements that significantly impact the practice of interventional pulmonology [1]. Bronchoscopy is essential for the diagnosis and staging of lung cancer by assessing the primary tumour (T descriptor) and lymph node involvement (N descriptor). The 9th edition maintains the T descriptors from the 8th edition but introduces pivotal modifications to the N and M categories. N2 lymph nodes are now divided into N2a (involvement of single ipsilateral mediastinal or subcarinal nodal station) and N2b (involvement of multiple ipsilateral mediastinal nodal stations with or without involvement of the subcarinal nodal station). T1N2a and T3N2a subgroups are now assigned to stage groups IIB and IIIA, respectively, whereas T2N2b is assigned to IIIB. Notably, N1 subcategories were not included in the current classification, despite evidence of a survival difference between N1a and N1b in pathological staging, but no significant differences in clinical staging [2, 3]. In addition, the M1c transcriptor was divided into M1c1 and M1c2 (single or multiple organ system metastases).

These changes enhance anatomical precision and align staging with contemporary therapeutic approaches, particularly as treatment strategies become increasingly differentiated based on nodal burden.

A precise mediastinal staging in lung cancer patients is crucial, as nodal involvement has an impact on therapeutic strategies and prognosis [1, 4]. Thereby, the combination of endobronchial ultrasound guided transbronchial needle aspiration (EBUS‐TBNA) and endoscopic oesophageal ultrasound guided fine needle aspiration (EUS‐FNA) is recommended for mediastinal staging as the first step in the following patient groups [5]: (1) enlarged and/or fluorodeoxyglucose (FDG)‐avid mediastinal and/or ipsilateral hilar lymph nodes on positron emission tomography (PET), (2) FDG‐negative primary tumours, (3) tumour size ≥ 3 cm. When an indication for mediastinal staging is given, systematic staging is recommended. The definition of systematic staging varies in the literature, while the most common recommendation is to sample at least three different mediastinal nodal stations (4R, 4L, and 7) [5, 6]. Prospective and retrospective studies demonstrated the superiority of complete mediastinal staging versus targeted staging where only FDG‐avid nodes are sampled [7, 8]. However, it must be considered that patient enrolment for these studies was conducted 10 years ago and that PET‐CT scanners are now assumed to be more sensitive. Thus, the sensitivity of targeted staging would certainly need to be re‐evaluated in clinical trials. The general approach to the staging examination is to sample the highest station lymph nodes first, progressing through the N2 and N1 lymph node stations to avoid tumour cell contamination and thus falsely upstaging.

For endosonographic staging, the subdivision of N2 into N2a and N2b raises the question of whether the EBUS needle should be changed between different N2 positions to prevent tissue contamination and false declaration of a multiple N2 (N2b) involvement. In a global survey on current practices of endosonographic N2 staging following the 9th edition of the TNM Classification [9], 605 bronchoscopists responded to the question ‘When puncturing two lymph nodes in the N2 category, do you change the needle between the punctures?’ as follows: 27.1% of respondents did not answer, 5.5% answered ‘yes’, 57.4% answered ‘no’, and 10.1% answered ‘sometimes’. The question ‘Do you believe it is important to change the needle between the punctures of two N2 lymph nodes?’ was answered by 20.7% of respondents with ‘yes’, 33.2% of respondents with ‘no’, and 17.4% of respondents with ‘sometimes’. So far there is a lack of evidence to answer this important question sufficiently and economic considerations hinder the implementation of routine needle changes [10]. One prospective study that evaluates the necessity of changing the needle between different N2 positions is ongoing and hopefully answers this important question (NCT07418450).

Cryo‐EBUS obtains larger tissue samples with preserved architecture, enhancing diagnostic yield, particularly for benign diseases, lymphomas and lymphoproliferative disorders [11]. While cryo‐EBUS shows no significant improvement over conventional EBUS‐TBNA for lung cancer, it enables molecular testing in 97% of samples versus 79% with EBUS‐TBNA alone [11]. This advantage is particularly relevant in never‐smoking lung cancer patients or in Asian populations where lung cancer is characterized by a high percentage of targetable mutations [12], requiring adequate tissue for comprehensive molecular characterization.

Recent technological innovations in interventional pulmonology improve the possibilities of diagnosing lymph nodes. While the N1 subdivision is not incorporated in TNM 9, and sampling lymph node stations 5 and 6 is currently not routinely performed for staging, peripheral lymph nodes cannot always be clearly distinguished from tumours or second carcinomas, which makes evaluating these lesions valuable. As it is not feasible to sample lesions that are distant from central airways using conventional EBUS, two new technologies have been developed to close this diagnostic gap.

The new thin convex‐probe EBUS has a smaller tip diameter (5.9 mm vs. 6.9 mm), greater upward angulation (170° vs. 160°), and improved manoeuverability, enabling access to peripheral airways and segmental lymph nodes that were previously unreachable with conventional EBUS. This platform demonstrates superior accessibility compared to conventional EBUS and successfully enables cytological assessment of subsegmental lymph nodes, facilitating more precise N1 staging [13].

Robotic‐assisted bronchoscopy (RAB) represents another transformative advancement, achieving high diagnostic yields of 80.4% for peripheral pulmonary lesions with an excellent safety profile [14]. Not only can pulmonary nodules be sampled by RAB, but also ‘peripheral’ lymph nodes. There are successful reports of sampling subaortic lymph nodes (station 5) and paraaortic lymph nodes (position 6). The robotic catheter was placed in the left upper lobe to sample those lesions. Sampling peripheral lymph nodes at stations 12, 13, 14 is also feasible based on clinical experience [15].

The 9th TNM edition's emphasis on nodal station enumeration aligns with emerging evidence that the number of stations involved provides superior prognostic discrimination compared to location‐based classification alone. Future iterations may further integrate station count with anatomical location, potentially creating hybrid classification systems that optimize prognostic accuracy. The role of peripheral lymph node stations in this framework remains under investigation, with data suggesting their routine assessment may enhance staging precision.

## Funding

The authors have nothing to report.

## Conflicts of Interest

J.M.B. received lecture and consulting fees from Intuitive Surgical Inc., Astra Zeneca, Boehringer Ingelheim, Olympus, Pulmonx, microtech; and research support from Beatrice von Hardenberg Stiftung, Thoraxstiftung Heidelberg, Günther‐Labes und Helene‐Heim‐Stiftung. All activities are outside the submitted work. D.G. received lecture fees from Pulmonx, Olympus, Erbe, AstraZeneca, MSD, Böhringer Ingelheim. All activities are outside the submitted work.
